# A rare case of a testicular lesion related to hand, foot, and mouth disease

**DOI:** 10.1002/iju5.12721

**Published:** 2024-04-01

**Authors:** Ayaka Igarashi, Yutaka Takezawa, Yuki Ozawa, Tomomi Saito, Hiroshi Nakayama, Takeaki Makino, Toru Etsunaga, Yoshitaka Saito, Mikio Kobayashi

**Affiliations:** ^1^ Department of Urology Isesaki Municipal Hospital Isesaki Gunma Japan

**Keywords:** diagnostic imaging, hand foot and mouth disease, testis, viral infection

## Abstract

**Introduction:**

Hand, foot, and mouth disease generally occurs in children. In rare cases, hand, foot, and mouth disease affects the testicles.

**Case presentation:**

A 29‐year‐old man presented to our emergency department with testicular pain for several days after the onset of hand, foot, and mouth disease. Ultrasonography revealed hypoechoic mass‐like areas in the right testis. A mild inflammatory response was noted, tumor markers and urinary data were normal, and tests for infection were all negative. Antibiotics were initiated and ultrasonography was performed in every subsequent examination. Testicular pain disappeared 6 months later.

**Conclusion:**

We encountered a rare case of a testicular lesion related to hand, foot, and mouth disease that was successfully treated. The careful selection of treatment for testicular pain and scrotal enlargement in young adult males, such as surgery and symptomatic treatment, based on their medical history and laboratory findings, is important.

**Abbreviations and Acronyms**
AFP: alpha‐fetoprotein
CRP: carbon reactive protein
CVA6: Coxsackievirus A6
HBs antigen: hepatitis B surface antigen
HCG: human chorionic gonadotropin
HCV: hepatitis C virus
HFMD: hand, foot, and mouth disease
HIV: human immunodeficiency virus
LDH: lactate dehydrogenase
MRI: magnetic resonance imaging
PCR: polymerase chain reaction
pH: potential hydrogen
RPR: rapid plasma regain
TPHA: treponema pallidum hemagglutination
WBC: white blood cell

**Keynote message**

Testicular pain in male adults may suddenly occur due to various onsets. When ultrasonography reveals a tumorous lesion or nodule in the testis, it needs to be differentiated from a testicular tumor. The careful selection of treatment options for young adult males with testicular pain/scrotal swelling, such as surgery and symptomatic therapy, based on an interview on their medical history or various examination findings is important to avoid excessive invasion such as orchidectomy.

## Introduction

HFMD is an infectious disease in which droplet, contact, or fecal‐oral infection with CVA6, CVA10, and CVA16 or enterovirus 71 induces vesicular exanthema measuring 2 to 3 mm in diameter on distal parts of the extremities, such as the dorsal palms and soles and oral mucosa. We herein report a 29‐year‐old male with a testicular lesion related to HFMD who received symptomatic therapy and conducted a literature review.

## Case presentation

A 29‐year‐old male developed right testicular pain several days after the onset of HFMD. He took an analgesic, which was ineffective. Therefore, he presented to the Emergency Outpatient Unit of our hospital the next day. His medical/family history was not contributory and there was no episode of trauma. A physical examination showed a body temperature was 36.5°C, right‐dominant testicular pain, and an ambulatory status. Partial swelling of the right scrotum without induration was noted.

Blood biochemical findings were as follows: WBC, 10 200/μL; CRP, 3.21 mg/dL; LDH, 349 U/L (normal range: 119–220 IU/L); AFP, <1.3 ng/mL (normal range: ≤9.6 ng/mL); and HCG, <2.0 mIU/mL (normal range: ≤10.0 mIU/mL). Qualitative RPR test, qualitative TPHA test, HBs antigen, HCV antibody, and HIV antibody tests were all negative. Urinalysis revealed the following findings: pH, 6.5; specific gravity, 1.014; occult blood (−); ketone body (−); nitrite (−); leukocytes (−); urinary protein (−); bilirubin (−); urobilinogen (±); and glucose (−). The following urinary sediment findings were obtained: RBC, <1/HPF; WBC, <1/HPF; bacteria (−); and atypical cells (−).

**Fig. 1:**
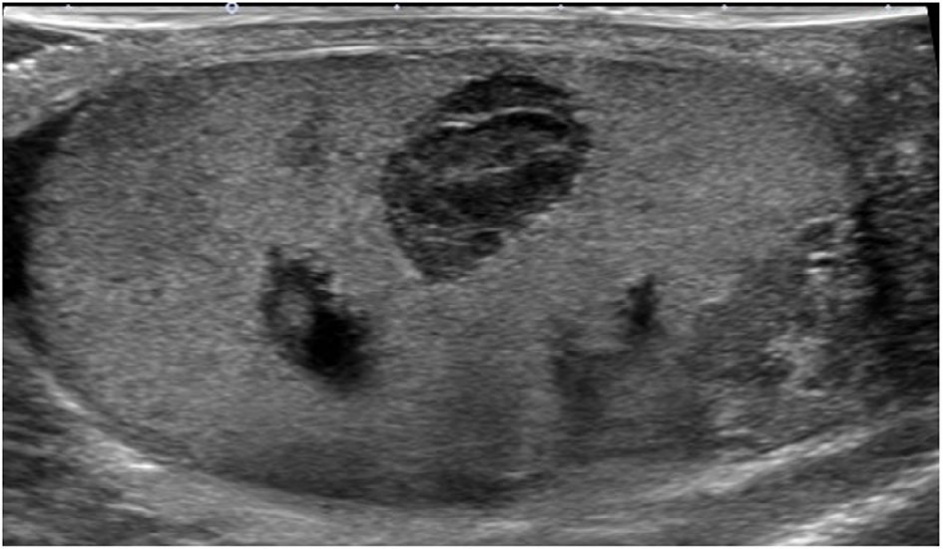
Ultrasonography image of the right testis on the first visit. Testis size was 47 × 21 × 22 mm. Several nodules with an unclear border were detected in the upper and lower poles.

Ultrasonography showed no laterality in testis sizes (right: 47 × 21 × 22 mm, left: 42 × 24 × 24 mm) or blood flow disturbances. Several nodules with an unclear border were detected in the upper and lower poles of the right testis. Internal echo was slightly heterogeneous in the left testis and there were no nodules (Fig. 1).

**Fig. 2:**
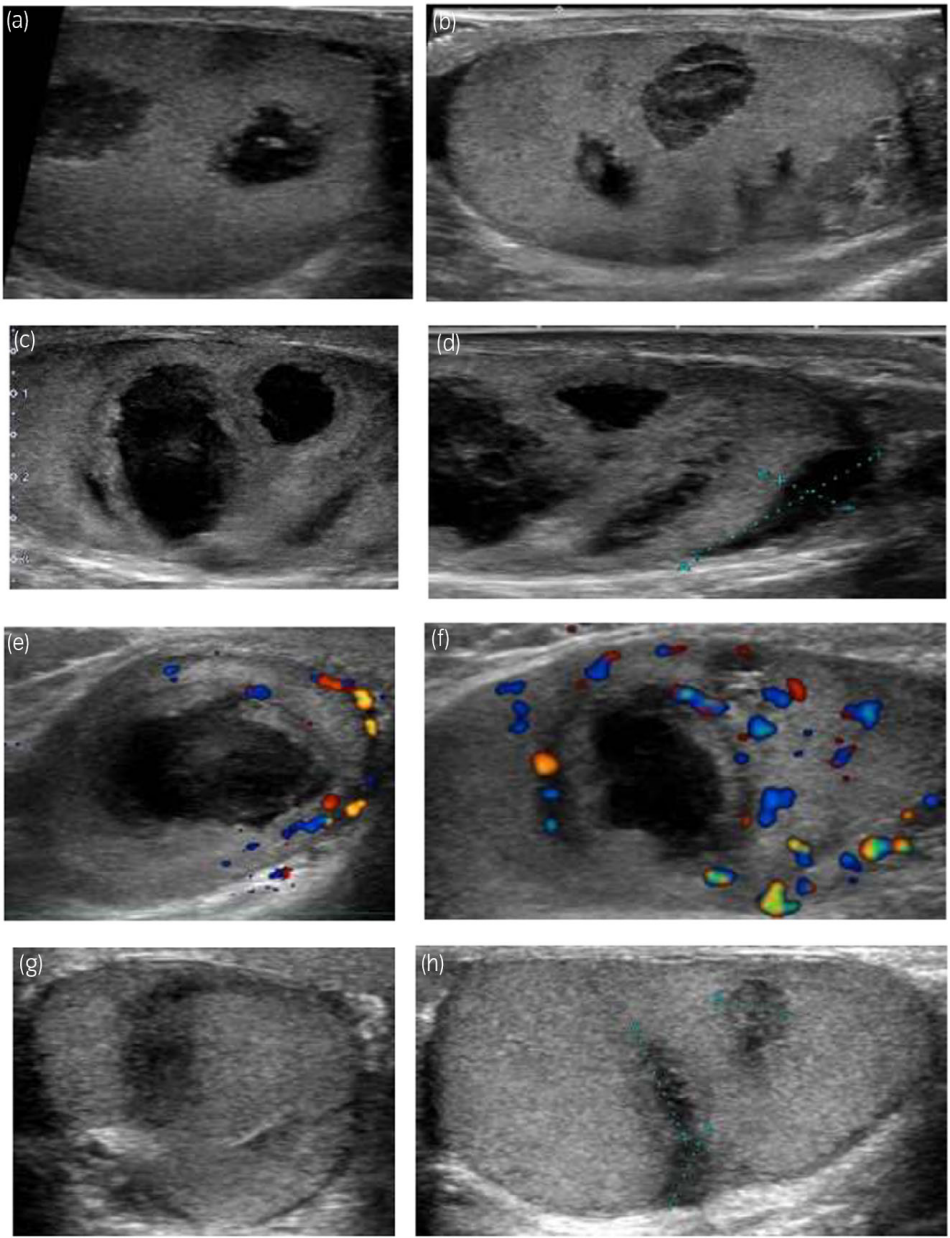
The time‐elapse ultrasonography images of the right testis. Nodules disappeared 6 months after the first visit. The left‐side images of each line are transverse sections, and the right‐side images are sagittal sections. (a,b) Day 1; (c,d) Day 5; (e,f) Day 13; (g,h) Day 182.

The patient was orally administered cefuroxime for 7 days to reduce inflammation. Testicular pain recurred 13 days after the initial consultation and the patient consulted our hospital. Partial swelling of the right scrotum with induration was observed, but with a reduction in the hypoechoic area; therefore, an analgesic was prescribed. Thereafter, pain did not recur, and normal findings were observed in a 6‐month follow‐up. The course of ultrasonography findings is shown in Figure 2.

## Discussion

Orchitis related to mumps virus infection accounts for 20% to 30% of cases of testicular pain in adult males. However, testicular pain and scrotal swelling have also been reported in some patients with CVA6, adenovirus, enterovirus, or COVID‐19 infection.^1^, ^2^ Di Lella *et al*., a lesional PCR swab was positive for coxsackievirus from the patient's papulovesicular rash on his palms, nose and feet.^2^ Vuorinen *et al*. described a CVA6‐infected patient who simultaneously developed HFMD and epididymitis and reported that antimicrobial administration attenuated these conditions.^3^ Epididymitis is characterized by swelling of the epididymis and increased blood flow on color Doppler ultrasonography.^4^ Tarantino *et al*., grayscale sonography of the scrotum can show 1 or more indicative features in patients with epididymo‐orchitis of mumps virus infection: focal or diffuse enlargement of the epididymis with an anomalous echo texture, an enlarged dedimus with a heterogeneous echo texture, hydrocele, and scrotal skin thickening. The presence of hyperemia on color Doppler ultrasonography was the only sonographic sign of inflammation in 40% of cases of orchitis.^5^ Singh *et al*., 30%–50% of patients showed testis atrophy after mumps orchitis. Although testicular cancer is not casually associated with mumps epididymo‐orchitis, it has been reported in men with testicular atrophy secondary to mumps.^6^ In the present case, the ultrasonography showed no laterality in testis sizes or blood flow disturbances in color Doppler, internal echo was slightly heterogeneous in the left testis. After 182 days of the first presentation, each testicular atrophy was not seen in ultrasonography. If ultrasonography reveals a tumorous lesion or nodule in the testis, it needs to be differentiated from a testicular tumor in adults. In these cases, early orchidectomy must be considered, assuming lesion metastasis or disease progression.^3^ In the present case, diseases to be differentiated initially included epididymitis, a testicular tumor, mumps orchitis, testicular torsion, and appendiceal torsion based on the age of the patient at the time of onset, symptoms, and physical findings. There was no finding to indicate urinary tract infection. The patient was negative for tumor markers and the lesion was painful; positive findings for the above diseases were insufficient. Since some infectious diseases induce a mild inflammatory response, we selected antibiotic administration and symptomatic therapy. Although a few case reports of testicular lesions related to HFMD have been published, this condition is extremely rare. Hurtt *et al*. described two patients with testicular pain after the onset of HFMD, including a 36‐year‐old male who selected and underwent surgical extirpation, namely, orchidectomy, through sufficient counseling on the possibility of early progression or metastasis in the case of a malignant tumor or fertility loss after orchidectomy. In the two patients, pathological findings included inflammatory changes, fibrosis, and hemorrhagic changes. There was no tumor or solid component.^1^ The follow‐up of testicular lesions is not recommended due to the possibility of a malignant tumor or the risk of metastasis. It is very difficult to tell the difference between malignant and benign conditions. If the symptomatic therapy is selected, both testicles should be monitored carefully. If viral infection is considered to be an etiological factor, follow‐up by ultrasonography needs to be continued at short intervals to avoid patient anxiety or unnecessary surgical invasion through sufficient patient‐shared decision‐making.^7^ In the present case, ultrasonography was performed frequently; therefore, it was possible to serially evaluate the condition. Furthermore, the patient received symptomatic therapy, which ameliorated symptoms in the absence of excessive invasions, such as orchidectomy.

## Conclusion

We encountered a patient in whom symptomatic therapy for testicular pain after the onset of HFMD resulted in amelioration. In the present case, diseases to be differentiated included a testicular tumor requiring early orchidectomy; however, the findings obtained were not typical for various diseases, and, thus, symptomatic therapy was selected. The careful selection of treatment options for young adult males with testicular pain/scrotal swelling, such as surgery and symptomatic therapy, based on an interview on their medical history or various examination findings, is important.

## Author contributions

Ayaka Igarashi: Conceptualization; data curation; writing – original draft. Yutaka Takezawa: Conceptualization; supervision; writing – review and editing. Yuki Ozawa: Data curation; investigation. Tomomi Saito: Data curation; investigation. Hiroshi Nakayama: Data curation; investigation. Takeaki Makino: Data curation; investigation. Toru Etsunaga: Data curation; investigation. Yoshitaka Saito: Data curation; investigation. Mikio Kobayashi: Conceptualization; supervision; writing – review and editing.

## Conflict of interest

The authors have no funding to declare for this article.

## Approval of the research protocol by an Institutional Reviewer Board

Not applicable.

## Informed consent

Oral informed consent for publishing this case report was obtained from the patient.

## Registry and the Registration No. of the study/trial

Not applicable.
